# Average booking curves draw exponential functions

**DOI:** 10.1038/s41598-023-42745-3

**Published:** 2023-09-22

**Authors:** Masaru Shintani, Ken Umeno

**Affiliations:** 1 Kyoto University, Graduate School of Informatics, Kyoto, 606-8501 Japan; 2 Research and Development Department, FORCIA, Inc., Tokyo, 160-0022 Japan

**Keywords:** Statistical physics, Scientific data, Civil engineering, Applied mathematics

## Abstract

The booking curve time series in perishable asset industries, including hotels, has been studied to manage a demand-supply condition or revenue management (RM). However, due to changing times, e.g., economy and technology, many RM practitioners have put their efforts into catching on to peoples’ booking pattern shifts, representing macroscopic changes in booking curves. We investigate macroscopic aspects of booking curves with actual sales data across six properties in the hotel and car-rental industries for two years, considering the difference in the economic environment characterized before and during the COVID-19 epidemic. We explain a new cross-industry and cross-economic-environment universal statistical law: average booking curves draw exponential functions (the ABCDEF law). We provide a basis for the ABCDEF law from three perspectives; data validation, modeling in the statistical physics framework, and empirical justification for the causality of the model. The ABCDEF law derives informative statistics to quantitatively measure peoples’ buying behavior even in time or society changes, which is expected to contribute to management in various industries.

## Introduction

This study explains a new universal statistical law, recently discovered, regarding the booking curves time series.

For over 30 years, the perishable assets industry, comprising hotels and airline ticketing, has utilized revenue management (RM) to manage inventory over a finite sales horizon to maximize profits^1–5^. The application fields have been diverse^6,7^ such as car rentals^8,9^, golf^10,11^, cruise lines^12,13^, railway tickets^14^, theme parks^15^. The cumulative time series of bookings represent a booking curve. It has traditionally been a primary subject in RM efforts, as it directly relates to occupancy forecasting, which is a critical issue in RM^2,5,6^. In the case of airline tickets, a 20% improvement in forecasting accuracy has been reported to increase sales by 0.5–3%^3^. Therefore, the use of booking curves for forecasting in RM has been widely studied by many researchers and professionals in the perishable assets industries. This has led to the development of several advanced and highly accurate forecasting models^4,9–11,14,16–21^, including exponential smoothing^22–25^, stochastic process models^3,26^, generalized linear mixed models^27^, and methods utilizing neural nets that incorporate seasonal and day-of-week trends into their parameters^28,29^.

However, mistiming the launch of RM measures due to people’s booking pattern shifts is an issue that RM professionals face. Since the fundamental idea of RM is to sell the right product at the right price and at the right time, RM professionals need to understand how the timing of people’s bookings changes with the shifts in the demand-supply environment to ensure the effective use of advanced forecasting. Conjoint analysis for managers indicates that the factor most likely to affect performance in pricing, which is the most typical measure in RM, is appropriate timing (that is, the booking window)^30^. Therefore, it is important to measure changes in booking curves quantitatively, that is, the booking window shift, to ensure the performance of RM.

In general, the causes of changes in booking curves can be divided into micro factors and macro factors^18,28^. Micro-environmental factors have been studied and incorporated into daily forecasting models, including seasonality, trends in a day of the week, pricing, and promotions. Macro-environmental factors have posed a challenge for RM professionals when considering the appropriate timing for taking RM measures and include factors, e.g., the development of technologies, economic conditions, and people’s preferences.

Hotel and airline ticketing industries have reported that a complex (that is, “grow” and “shrink”^28^) booking window shift has occurred due to the spread of IT technologies, and the booking window has shrunk due to extreme delays in decision-making during the COVID-19 pandemic^31,32^. To find appropriate opportunities to sell products at the right price and timing, RM professionals have been required to analyze these complex booking window shifts, which can occur both gradually and rapidly due to macro-environmental factors. In other words, it is essential to have information to understand macroscopic changes in booking curves of each industry and property due to the changing times to take full advantage of sophisticated forecasting algorithms. In this study, therefore, while we focus on booking curves, which use “days” units in conventional forecasting modeling, we choose “months or a year” as a unit of time to evaluate the booking window shift. By doing so, we provide an explanation that summarizes the effect of changes in the demand-supply environment on people’s booking patterns.

Based on this background, we analyzed actual sales data provided by six properties—three each in the hotel and car rental industries—, whose data acquisition period is for two years, including the periods before and during the COVID-19 pandemic. We studied the average booking curves, a new statistical series defined as an average time series of booking curves over a period. We explain a new universal statistical law characterized by the exponential function, which we called the ABCDEF (average booking curves draw exponential functions) law.

## Overview of ABCDEF law

This section presents an overview of the ABCDEF law. A booking curve time series exists in capacity-constrained assets (perishable assets) industries such as hotels, airline tickets, car rentals, golf, cruise lines, and theater seats. It is defined, in this study, as a cumulative time series of bookings toward a deadline over a finite sales horizon.

Strictly speaking, there are two types of definitions of booking curves. In this study, we use a “net” demand-based booking curve that reflects only the actual demand consumed^4,17,22,23,25^. The other booking curve is represented as a “gross” demand-based booking curve (on-hand-based booking curve generally). The latter is often used in studies for RM^3,10,12,16,18,19,24,26,28^. Each definition is categorized based on whether or not canceled bookings are considered in the time series; the former does not include them (that is, aggregate only consumed bookings), and the latter includes them (“gross” shows the original data at the time). When *q*(*t*) is the number of bookings that are received at *t* days before the deadline, the (net demand-based) booking curve is expressed as a cumulative time series of *q*(*t*). In other words, the booking curve *X*(*t*) at *t* days before the deadline is defined as follows:

1 $$\begin{aligned} X(t) \overset{\text {def}}{=} \int _t^{\infty } q(s) ds. \end{aligned}$$

**Figure 1:**
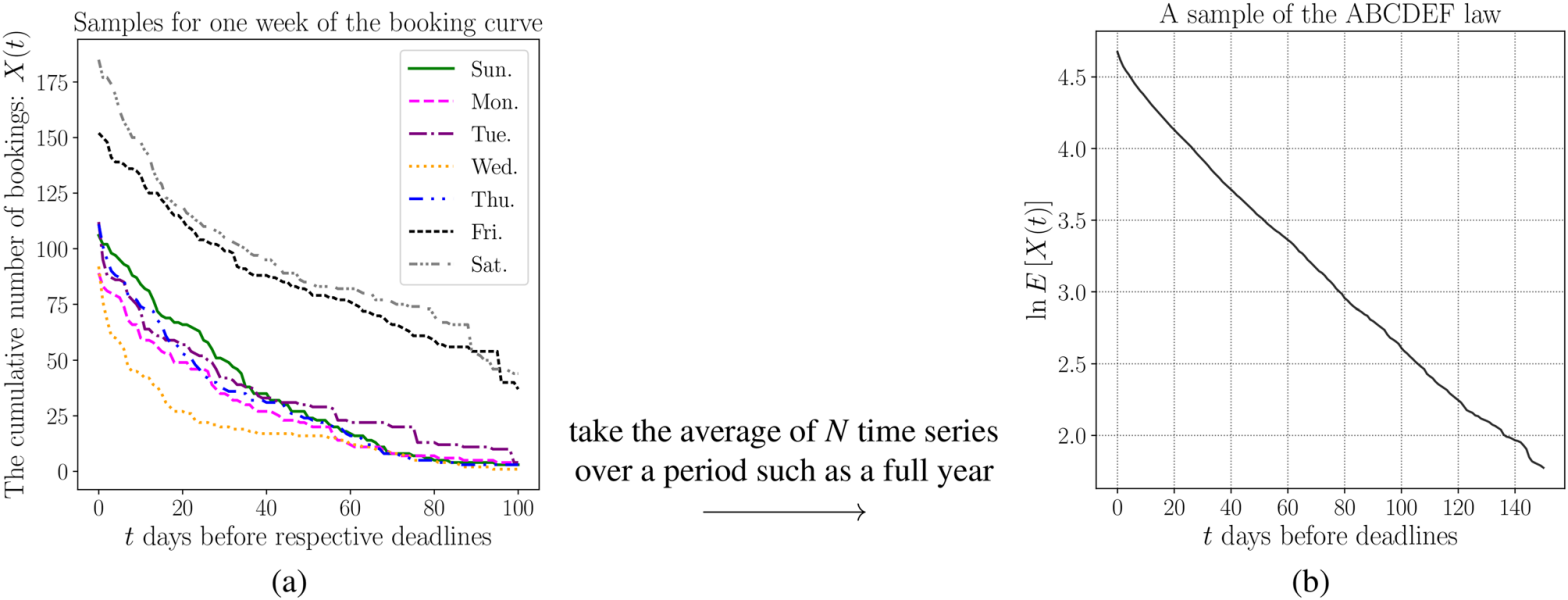
An outline of the ABCDEF law. Figure (**a**) shows the (net demand-based) booking curves for a hotel for one week in 2019. Since demand varies depending on the day of the week and other factors, the transitions of booking curves have different trends. Figure (**b**) shows an average booking curve time series with a logarithmic scale derived from data for the year 2019 in the same property. The straight line with a logarithmic scale represents the exponential law of the average booking curve.

We study the characteristics of booking curves *X*(*t*). Here, we introduce a statistical series based on booking curves, that is, the average booking curve, which is defined as the average statistical time series of *N* bookings curves over the observation period, such as months or a year, and is defined as follows:

2 $$\begin{aligned} \mathbb {E} [X (t;n)] \overset{\text {def}}{=} \frac{1}{N}\sum _{n=1}^N X(t; n), \end{aligned}$$

where *n* represents a business day. The ABCDEF law derived in this study shows that an average booking curve (2) is universally characterized by the exponential function as follows:

3 $$\begin{aligned} \mathbb {E} [X (t;n)] \simeq A \exp (-\beta t), \end{aligned}$$

where *A* is a parameter that depends on the quantity demanded and $\beta$ is a parameter that indicates the demand-supply environment surrounding the property. Figure 1 shows an example of the law provided from actual data.

As for the implications of the two parameters, *A* is mainly determined by the initial inventory of the product and the magnitude of demand. While the parameter $\beta$ indicates the demand-supply environment surrounding the property, it also illustrates patterns in people’s booking horizons, which can be observed based on the shape of the function. These characteristics in $\beta$ are two sides of the same coin. For example, the value of $\beta$ becomes larger when the ratio of advance booking increases, and vice-versa with the ratio of last-minute bookings. In other words, a larger value is created by high-demanded conditions such as vacations or discounts, where consumers tend to book in advance. In contrast, a smaller value is created due to high percentages of unplanned bookings, such as on-the-day actions, delayed bookings by strategic consumers for last-minute discounts, or missing reservations due to price competition with neighboring facilities.

Note that, while the values of these two parameters, *A* and $\beta$, differ for each property as expected, they also differ in the same property according to the time shift. In this study, therefore, our argument of “universality” in the ABCDEF law is based on the uniqueness of each industry and each demand-supply environment.

We justify the universality of the ABCDEF law from the following three perspectives: data validation, modeling in the statistical physics framework, and empirical justification for the causality of the model.

The data validation section shows the two-year actual sales data and fitting based on Eq. (3) for each property. We investigate by considering the difference in economic conditions, in addition to the difference in properties: we divided the data acquisition periods into groups according to before and during the COVID-19 epidemic. In the modeling section, we explain that an average booking curve follows an exponential function under a specific requirement for the demand-supply environment surrounding the property. We call the requirement a homogeneous demand-supply bath assumption (HDSBA: Section “A universal statistical law: average booking curves draw exponential functions” in details). In the empirical justification section, to provide a basis for the causality of the model, we discuss whether the exponential function is derived when the HDSBA is satisfied. Specifically, we introduce a quantitative definition of a homogeneous “degree” of the environment, which characterizes the HDSBA. We investigate a correlation between the homogeneous “degree” and an exponential property, described as fitting deviation by exponential functions and average booking curves.

The ABCDEF law, justified from the above three perspectives, provides booking curves with its usefulness besides daily forecasting in RM. New statistics based on the averaged property of booking curves can provide more information about people’s booking timing patterns, which change depending on various factors, e.g., the economic environment, preferences, pricing, and promotions. Thus, the statistics facilitate RM professionals to take measures at appropriate timing.

## Literature review

Our literature review mainly focuses on booking curves and windows, which are closely tied to each other because a booking curve only represents the cumulative time series of the number of bookings over a booking window. Thus when booking windows shift, the time-series patterns in booking curves also change.

### History

A booking curve has been used as one of the most important elements in forecasting for RM whose objective is to maximize revenue over a finite sales horizon in the perishable assets industries. As for the history of RM, it originates from yield management (control an allocation for finite inventory) in the airline industry in the United States due to the deregulation of pricing in the 1970s^1,2^. Subsequently, yield management derives RM with the fundamental idea of the right product at the right price and at the right time. RM has become part of public research themes since the late 1980s, and many models and business instance reports have been illustrated over the past 30 years in various perishable assets industries^3–15,33^. A study on airline tickets among them showed that a 20% improvement in the accuracy of quantity demand forecasting led to a 0.5–3% increase in revenue. Based on such arguments, booking curves, which are directly related to occupancy forecasting, play an important role in the management^3^. In the past, RM research was difficult to expand because actual sales data could not be obtained due to confidentiality issues^34^, however, the commonly available data for analysis are increasing^35^.

Booking curves represent a cumulative time series of bookings, and has two types defined in previous studies; “gross” (in general on-hand) demand-based booking curves and “net” demand-based booking curves. They are divided by types to compile cancellation data. The former shows actual observation numbers at the time; in other words, it includes lost bookings after the observation day due to cancellations. The latter does not include canceled bookings; all booking data to be recorded are finally presented. The former is generally used in RM studies because it has more realistic situations for forecasting. In contrast, the latter is often used to estimate imaginary demand that might have been achieved without the constraints of limited inventory in case of selling out. The commonalities of both booking curves are used for predicting the (actual or imaginary) number of bookings at the deadline based on historical or current dynamic time-series data.

As for forecasting models, various ones have been proposed using booking curves or booking processes. The most traditional and representative forecasting model is the exponential smoothing (including its expansions)^12,16,22–25,36–38^. In recent studies, various approaches, such as stochastic process models (including Poisson processes, negative binomial processes, generalized linear mixed models,), neural nets, pick up algorithms, and advance booking models have been proposed^3,4,10,12,16,18,19,26,28^. Since some of them are versatile, they spread in various perishable assets industries with some vertical advances such as dealing with the peculiar parameter in each field. While various sophisticated forecasting models using the booking curve have been developed, it has become clear that the utility of booking curves has mainly been biased towards “forecasting” and has few other purposes in any industry.

### What affects booking curves

A macroscopic change in booking curves and people’s booking window shifts are two sides of the same coin. In general, what influences booking window shifts are divided into macro factors, such as technology developments and changes in economic conditions, and micro factors, such as seasonality, day-of-the-week trends, pricing, sales channel allocations, and promotions^18,28^. In particular, IT technologies, one of the macro factors, e.g., the adoption of online agencies, mobile APPs, and RM operation systems, made it possible for consumers to make bookings anytime and anywhere and enabled strategies such as pricing for RM professionals. Thereby, IT technologies resulted in booking window shifts with complex characteristics like “grow” and “shrink”^28^ through the evaluation of changes in an average lead day of bookings, which is used to measure booking window shifts. For example, it is reported that high average lead days were observed for vacations, and low average lead days were caused by delayed bookings by strategic consumers for last-minute discounts that caused unfulfilled inventories^28^. The COVID-19 pandemic also caused booking window shifts. Various industries, including airline ticketing, reported that people’s booking windows were delayed due to uncertainties such as anomalous infection risks^31,32^.

The establishment of models to provide enough information regarding people’s booking pattern shifts, which result in macroscopic changes in booking curves, is expected to maintain revenue performance.

## The data analyzed

We used a variety-rich dataset on booking curves provided by several firms, for which we are grateful. The data was collected for a two-year period, including before and during the COVID-19 pandemic. This data is sufficient to allow us to investigate across not only multiple fields but also multiple economic environments.

This section presents a description of the data analyzed in terms of the following aspects: property information, actual sales data, and periods divided according to economic environment shifts.

### Industries, properties, and sales data

Table 1 shows the information on the properties from which the acquisition data was provided.

**Table 1:** Profiles for six properties in two industries which provide the actual sales performance data.

Property	Location	Region	Characteristics of services
Hotel (property I)	Tochigi	Regional city	Resort hotel
Hotel (II)	Osaka	City	Business hotel
Hotel (III)	Kyoto	City	Business hotel
Car rental (IV)	Island Ishigaki	Resort	Passenger car
Car rental (V)	Naha	Resort	Passenger car
Car rental (VI)	Island Miyako	Resort	Passenger car

Each property is located in Japan and is dispersed into areas with different economic characteristics, such as cities, regional cities, and resorts. Following this, Table 2 shows a part of the sales data recorded in property I. The data constitution also holds for other properties. We have “whole” sales data; that is, it has all of the booking data coming in each property.

**Table 2:** A part of the actual sales data set in a property I.

Use date	Price	Rooms	Room type code	Booking date	Is canceled	Cancel date	Booking lead days
2019-10-01	48,008	2	W1	2019-09-02	True	2019-09-07	29
2019-10-01	32,184	1	Kid2	2019-09-02	False		29
2019-10-01	21,602	1	W1	2019-09-07	True	2019-09-16	24
2019-10-01	26,730	1	Kid1	2019-09-12	False		19

The column set of use date and booking date in each record enables us to aggregate booking curves.

Sales data were recorded by the “bookings” unit, that is, its originals contained who made a reservation, at what time, which product was chosen, how many were booked, at what price, and through which sales channel. We can access the data that describes when, how many, and at what price while considering confidentiality, which allows us to summarize data for booking curves. In addition, in properties I, IV, V, and VI, we can also access cancellation data, which have information regarding the canceled day in the same row of the booking records. Although cancellation data are not necessary to summarize the net demand-based booking curves, which is the subject of this study’s analysis, it can improve the quality of the data for future studies.

Note that, especially in property I, we did not aggregate booking curves from chartered days, defined as having more than 50% group travel customers, to exclude people’s extraordinary booking patterns. We can confirm whether a day is chartered or not in the repository.

### Economic environments

We divide the data acquisition period into groups because of some events which largely influenced the economic environments for every property. Since the COVID-19 crisis occurred all over the world, including in Japan, every property was economically affected. Expressing the period 2019 as “regular” demand, we regard the period 2020 as “irregular” demand. Then, we divide 2020 into three groups according to events that significantly changed the market condition or people’s mobility in the whole country. Table 3 shows definitions and characteristics of the period in 2019 and three periods in 2020.

**Table 3:** The information of periods divided in terms of economic environments.

Period	Time range	Remark for market conditions
2019 (’19)	Jan. 1–Dec. 31 (2019)	“Regular” demand
2020-1st (’20-1st)	Jan. 1–Mar. 31 (2020)	The first COVID-19 epidemic
2020-2nd (’20-2nd)	Apr. 1–Jul. 21 (2020)	A state of emergency
2020-3rd (’20-3rd)	Jul. 22–Dec. 31 (2020)	Tourism campaign

Our validation in this study is based on the comparison across these four periods.

There were three major events in 2020, according to the economic shifts due to the COVID-19 epidemic. From mid-January to February 2020, the first infection case was observed in Japan, subsequently, the number of cases increased, and restrictions on immigration and gatherings were imposed^39^. We define the first period as from January to March.

In April, the Japanese government declared a state of emergency, which continued until late May. The state of emergency resulted in a strong restriction on people’s mobility, with decreases up to 30% in urban areas^40,41^. On July 22, as the wave of the number of infected largely decreased, the Japanese government launched a tourism campaign called “Go-To Travel,” which assisted people in domestic travel by subsidizing up to 50% to encourage economic activities. This tourism campaign ended on December 28 due to the re-spread of COVID-19 infections^40,41^. Thus, we define the second and third periods as April 1 to July 21, and July 22 to December 31, respectively.

Here, we check the states of the economic environment in each property over the divided periods. Table 4 shows the relationship between the quantity demanded and the average lead day of bookings, which is a statistic to measure changes in the booking window shifts^28,31,32^, year on year in the same period.

**Table 4:** This table shows the changing ratio for the quantity demanded and the average of lead days of bookings, representing the outline of the demand environment.

	Quantity demanded			Average of lead days		
	’20-1st	’20-2nd	’20-3rd	’20-1st	’20-2nd	’20-3rd
Prop. I	0.74	0.30	0.76	0.95	0.24	0.63
Prop. II	0.58	0.08	0.23	1.58	0.09	0.15
Prop. III	0.42	0.06	0.55	1.05	0.19	0.27
Prop. IV	0.99	0.27	0.79	0.92	0.46	0.57
Prop. V	0.69	0.16	0.60	1.02	0.44	0.58
Prop. VI	0.70	0.22	0.92	1.02	0.53	0.60

The values are obtained regarding the result in the same period in 2019 as 1, respectively. Note that the quantity demanded is defined as $\frac{1}{N}\sum _{n=1}^N X(0; n)$, where *N* is the sum of business days in the period. An average of lead days is defined as $\frac{1}{N} \sum _{n=1}^N \left[ \frac{\sum\nolimits _t t q(t;n)}{\sum\nolimits _t q(t;n)} \right]$. In this table, the word property is abbreviated as prop.

The changes in quantity demanded had the following general trends in all properties; a slight or up to 50% decrease in 2020-1st, a significant decrease of up to 70–90% in 2020-2nd, and a general recovery with differences among properties in 2020-3rd, This seems to reflect the effect on mobility in each characteristic of the period, the COVID-19 first pandemic, a state of emergency, and the tourism campaign.

Besides, the average lead days of bookings also had mostly common trends across all properties; the same level as the previous year in 2020-1st, large decreasing fluctuations up to 50–90% reduction (shrink) in 2020-2nd, and in 2020-3rd, a slightly or decent recovery (grow) along with quantity demanded recovery, especially up to 60% in four properties I, IV, V, and V. The significant decreases in 2020-2nd, can be attributed to the tendency to delay decision-making due to heightened uncertainty^31,32^. Considering the changes in these two economic representative values, we confirm that the four periods, including 2019, are considerably different regarding the demand environment and people’s booking patterns. The following sections investigate the booking curves across properties and economic environments. Then, we use the four periods above as economically different periods.

## A universal statistical law: average booking curves draw exponential functions

### The data validation

**Figure 2:**
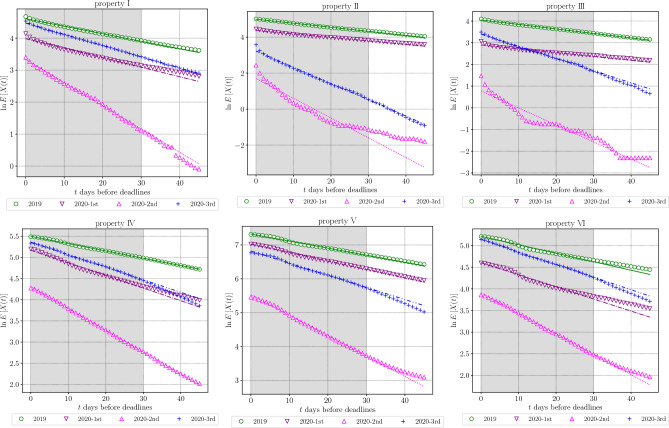
Average booking curves (marker plots) and fitted lines based on Eq. (3) for each property and economic environment. The fitting interval is $0\le t\le 30$ and is represented by the shaded part. The information for fitting lines is written in Table 5.

We compiled average booking curves (2) for each property listed in Table 1 and for each period in Table 3. Figure 2 illustrates a total of 24 series of average booking curves, which was constituted by a combination of the six properties and the four periods, and fitting lines based on Eq. (3). Table 5 shows the fitting parameters and the number of aggregated days.

**Table 5:** The fitting parameters *A* and $\tau (=1/\beta )$ are illustrated in Fig. 2.

	*A*: magnitude of demand				$\tau (=1/\beta )$: environment				*N*: aggregated days			
	’19	’20-1st	’20-2nd	’20-3rd	’19	’20-1st	’20-2nd	’20-3rd	’19	’20-1st	’20-2nd	’20-3rd
I	100.2	53.8	28.0	87.5	42.6	33.6	13.8	28.4	290	68	55	114
II	148.5	79.4	5.5	25.3	44.3	51.4	9.0	11.0	365	91	97	163
III	57.2	18.1	2.2	29.8	48.4	56.6	12.8	17.8	365	84	40	119
IV	224.3	175.8	74.4	212.8	57.9	33.5	19.4	33.7	365	91	112	163
V	1515.3	1124.8	251.7	915.5	48.0	40.1	16.6	28.0	365	91	112	163
VI	184.2	100.1	49.6	171.5	51.0	35.7	21.2	34.3	365	91	112	163

The intercept parameter *A* and the slope $\tau$ represent the magnitude of demand and peoples’ booking patterns depending on the environment. The number *N* is aggregated days for the average booking curve and corresponds to the number of business days. We adopt $\tau (=1/\beta )$, which is generally called a time constant in exponential decay functions, and analytically represents the day at which $1/e \times 100 \simeq 36.8$ percentile cumulative bookings are fulfilled when under the exponential law.

Although the parameters *A* and $\beta$ represent the magnitude of demand and the demand-supply environment surrounding the property, they show unique values for different periods, even at the same property. Confirming how the average booking curves draw exponential functions, in 2019, we see the coincidence in at least five of the six properties, excluding property VI. For the three periods in 2020, relatively large deviations from the exponential function were observed in the property II and III in the economic environment 2020-2nd. In addition, the following environments can be divided into cases with slightly more significant deviations; property I in 2020-1st, property III in 2020-1st, and property VI in 2020-1st.

Even if we evaluate these six patterns as not satisfying the exponential functions law, we can confirm the non-trivial coincidence in 75% (18/24) environments. In other words, the average booking curves are well characterized by exponential functions with unique parameters. These results imply a “universal” exponential law of average booking curves since it is a feature observed not only across industries such as hotels and car rentals but also across economic environments such as before and during COVID-19. We attempt to model the dynamics which derive the exponential nature of average booking curves in the following subsection.

### Modeling of exponential booking curves in the statistical physics framework

In modeling, we first conceptualize the relationship between the demand-supply environment of a property and the booking curve. Then we translate these relationships into a mathematical model.

**Figure 3:**
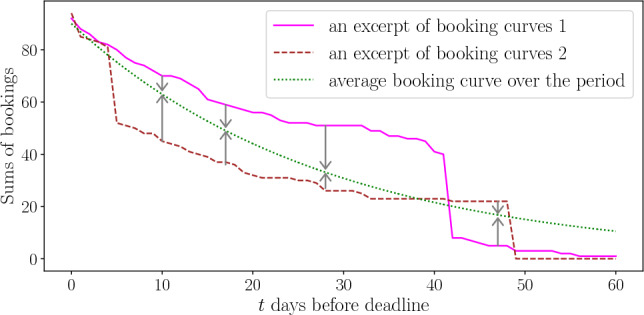
Due to the effects of revenue management, such as price changes, different build-up patterns can be observed even if almost the same number of bookings is reached. However, no bookings period or sharply rising patterns are smoothed by averaging. By averaging multiple time series, each arising from various demand-supply conditions as exemplified by the red and brown series, a smoothed curve (indicated by the green line) emerges, exhibiting less bias in its curvature.

A fundamental operation in revenue management involves appropriately adjusting supply and demand, including price, when sudden changes in the number of bookings are observed or when no bookings are made over a long period. For a given day, if too many bookings are made in advance, revenue managers may raise the price; for another day, if no bookings occur until the last minute, they may lower the price or increase advertising. Figure 3 presents an excerpt of booking curves for two days, illustrating that even on days that reach almost the same number of bookings, the temporal patterns of the time series differ due to changes in the demand-supply environment, including revenue management.

Given this, biased temporal changes in the number of bookings are smoothed in the average booking curve. Then the time series with the most negligible bias in the curve’s curvature can correspond to the one with the balanced demand-supply state.

**Figure 4:**
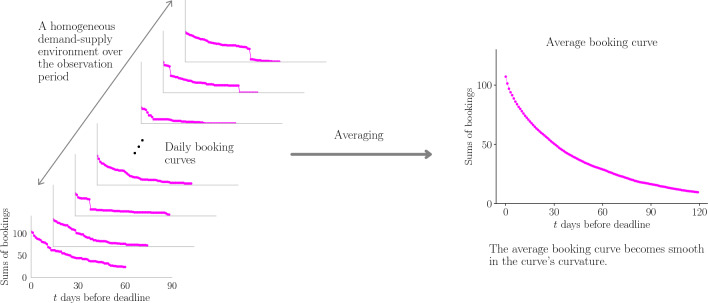
The overview of the conceptual model of HDSBA and average booking curves. When we give a homogeneous demand-supply environment, including efficient revenue management and the economic condition, over the business period, the average booking curve emerges as a smoothed series resulting from a balanced demand-supply state.

However, an important assumption is necessary to support this logic. That is, the environment surrounding the property must be an economic environment where revenue management can work efficiently. The necessity of this assumption can easily be understood by considering the opposite scenario. For instance, no matter how thoughtful measures try with revenue management, no bookings can occur, such as during a COVID pandemic or emergency declarations. Additionally, when a property is fully booked several months in advance without any revenue management, the modeling logic described above does not apply. Therefore, the condition assumed in the proposed model is called a “homogeneous demand-supply bath assumption (HDBSA),” which collectively refers to effective revenue management and an economic environment where they work. The reason for using the term “bath” will be detailed later in this chapter. Figure 4 illustrates the overview of the conceptual model.

Second, we then translate the above conceptual model into a mathematical representation. In particular, we consider the relationship between a booking curve and the average booking curve under the assumption of HDBSA.

Since a booking curve *X*(*t*) is a net demand-based booking curve, it builds up according to the deadline. Therefore, we define a booking curve of a day *n* using the following monotonically non-increasing function $\phi _n$.

4 $$\begin{aligned} X(t;n) \overset{\text {def}}{=} A_n\phi _n(t; \beta _{n,t}), \end{aligned}$$

where $A_n (> 0)$ is a reached occupancy constant, and $\beta _{n,t}> 0$ denotes a time-depending variable that reflects the build-up way of a time series. The function $\phi _n(t; \beta _{n,t})$ satisfies the following properties:

5 $$\begin{aligned} \begin{aligned} \phi _n(0; \beta _{n,0})&= 1, \quad \lim _{t\rightarrow \infty }\phi _n(t; \beta _{n,t}) = 0,\\ \phi _n(t; \beta _{n,t})&\ge \phi _n(t+1; \beta _{n,t+1}) \quad \text {for} \; \; \forall {t}\ge 0. \end{aligned} \end{aligned}$$

The parameter $\beta _{n,t}$ varies time-dependent even when comparing days that reach the same sales quantity (for example, in Fig. 3).

Here, we define the time series with the most negligible bias in the curve’s curvature in the conceptual model as follows with constant *A*, a function $\phi$ satisfying almost the same feature as Eq. (5), and $\beta _t$ of *t*:

6 $$\begin{aligned} \mathbb {E}[X(t;n)]&= \frac{1}{N}\sum _n A_n\phi _n(t;\beta _{n,t}) \\ &\overset{\text {def}}{\longrightarrow } A\phi (t;\beta _{t}) \quad \text {for} \; \; N \gg 1,& \end{aligned}$$

with

7 $$\begin{aligned} \begin{aligned} \phi (0; \beta _{0})&= 1, \quad \lim _{t\rightarrow \infty }\phi (t; \beta _{t}) = 0,\\ \phi (t; \beta _{t})&> \phi (t+1; \beta _{t+1}) \quad \text {for} \; \; \forall {t}\ge 0. \end{aligned} \end{aligned}$$

Then, according to the conceptual model, $A\phi (t;\beta _{t})$ has the most negligible bias in the curve’s curvature on *t*. In other words, the smoothed feature in $A\phi (t;\beta _{t})$ is equivalent to satisfying the following relationship on $\phi (t; \beta _t)$:

8 $$\begin{aligned} \frac{\phi (0;\beta _{0})}{\phi (1;\beta _{1})} = \frac{\phi (1;\beta _{1})}{\phi (2;\beta _{2})} = \cdots = \frac{\phi (t-1;\beta _{t-1})}{\phi (t;\beta _{t})} = \cdots . \end{aligned}$$

From Eq. (8) and $\phi (0)=1$, we obtain the following equation:

9 $$\begin{aligned}  \phi (t;\beta _{t})&= \phi (1;\beta _{1})\phi (t-1;\beta _{t-1}) \\&= \{\phi (1;\beta _{1})\}^2\phi (t-2;\beta _{t-2}) = \dots = \left\{ \phi (1;\beta _{1})\right\} ^t \quad \text {for} \; \; \forall {t}\ge 0.   \end{aligned}$$

Here, Eq. (9) is written as $\phi (t;\beta _{t}) = \exp (t \ln \phi (1; \beta _1))$; the expression represents that the effect of $\beta _t$ on $\phi (t;\beta _{t})$ is aggregated into $\exp (t \times \text {constant})$. Therefore, when we put $- \ln \phi (1; \beta _1) \equiv \beta \; (>0)$ because $0<\phi (1; \beta _1)<1 = \phi (0; \beta _0)$ for the assumed decreasing property of $\phi$, the function $\phi$ is uniquely determined as the exponential decay function $\phi (t; \beta ) = \exp (-\beta t)$. Note that the function $\phi$ satisfies $\frac{d\phi (t)}{dt} = -\beta \phi (t)$, which implies the property that an increase in the sums of bookings is proportional to the number of cumulative bookings. That implication is similar to “guests volume invites guests,” typified by the bandwagon effect and word-of-mouth^42,43^.

Third, we have one more point to consider; we explain the word “bath” in HDSBA. The modeling described above is structurally very similar to the relationship between a heat bath and a reaction system in thermodynamics, a branch of statistical physics^44^. If we call the system that handles the proposed model “booking curve dynamics,” it can be structurally corresponded to thermodynamics, as shown in Table 6.

**Table 6:** Relationship between booking curve dynamics and thermodynamics.

	Booking curve dynamics	Thermodynamics
Inner system	Business (ex. a hotel)	Test tube
Reaction	Sales	Chemical reaction
Surroundings	Demand and supply environment	Heat bath
Balancing approach	Revenue management	Exchange heat energy
Microstate	A daily booking curve	Energy state of one particle
Macrostate in the equilibrium	Average booking curve	Energy distribution
Expression for macrostate	$\exp (-\beta t)$	$\exp (-\beta E)$

**Figure 5:**
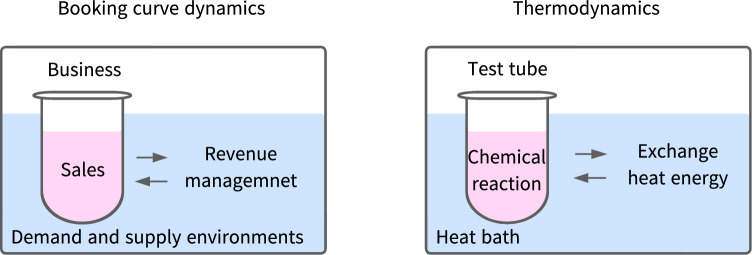
In thermodynamics, the heat bath contributes to creating an energy equilibrium state in the system, as shown in the right figure. We consider the analogy of thermodynamics for booking curve dynamics. Homogeneous demand-supply environments can result in a demand-supply equilibrium in average booking curves.

The visualized correspondence can be represented as shown in Fig. 5.

After comparing the objects in this way, we consider them from a statistical mechanics point of view. In other words, we discuss the relationship between the micro and macrostate and the process leading to the equilibrium state. Although it is impossible to precisely observe the microscopic energy state in thermodynamics, the macroscopic state at equilibrium can be explicitly expressed through stochastic formulation and its integration. Note that its explicit expression for energy distribution, the Boltzmann distribution, is the same exponential function $\exp (-\beta E)$, where $\beta$, in this case, is the inverse temperature of the heat bath, and *E* is the energy. Similarly, in booking curve dynamics, it is hard to formulate all of the microstates of booking curves (i.e., daily booking curves). However, examining their *average* behavior makes it possible to formulate the macro characteristics at equilibrium between supply and demand.

Finally, when we follow that the thermodynamic equilibrium results from the maximum entropy principle in thermodynamics, revenue management, a function to adjust demand and supply, can be regarded as an operation to maximize the “demand-supply entropy” of the business in the given economic condition.

For these reasons, the word “bath” derives from the heat bath concept, and HDSBA is analogous to the basic structure of thermodynamics.

### Empirical justification for causality

We empirically justify the causality of the above model. The key point of the proposed model is the causal relationship that the HDSBA results in the exponential law of average booking curves. To verify this causality in-depth, we define a degree of the cause and a degree of the result. In other words, we quantitatively provide a homogeneous “degree” of the demand-supply environment and a degree of similarity between fitted exponential functions and average booking curves. By doing so, we attribute the model’s causality to the degrees’ correlations. Note that that is strictly a correlation and not causality, but it significantly supports the validity of the consequence. Here, while we also use the two years data from 2019 to 2020 in this section, we compare annual data, not the periodized data in Table 3. That is because, as discussed in Section “The data analyzed”, the economic environments in 2019 and 2020 differ as regular vs. irregular. The objective is to find the emergence of a remarkable homogeneous “degree” of demand over the year by comparing annual data.

We focus on the quantity demanded, which reflects the changes in economic conditions, one of the compositions of the environment surrounding the property. We define an environment’s homogeneous “degree” by the magnitude of variation of the quantity demanded throughout the year, in other words, by the coefficient of variation. Thus, the small coefficient of variation denotes the small fluctuation in the quantity demanded and the high degree of homogeneous environment throughout the year. The mathematical definition of the coefficient of variation is as:

10 $$\begin{aligned} CV = \frac{\sigma _\text{q}}{\mu _\text{q}} \times 100 \ (\%), \end{aligned}$$

where $\mu _\text{q}$ and $\sigma _\text{q}$ are the mean and the standard deviation of seven days moving average time series of the quantity demanded for each year. We derive *CV* from the annual quantity demanded data for each year and each property.

**Figure 6:**
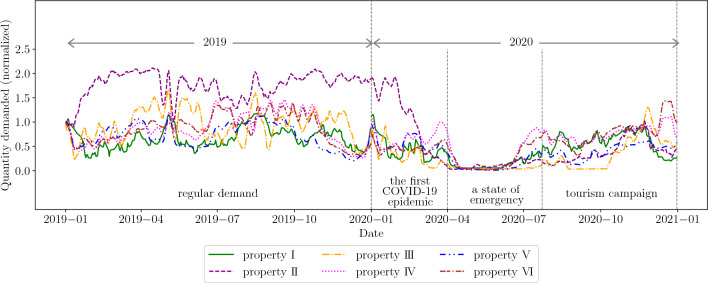
The time series in the figure show quantity demanded transitions. The values for each property are normalized by calculating the value on January 1st, 2019 as 1. These time series provide the coefficient of variations *CV*.

Meanwhile, the degree of similarity between fitted exponential functions and average booking curves is defined by the mean squared error *MSE* as follows:

11 $$\begin{aligned} MSE(N;t^*) = \frac{1}{t^*+1}\sum _{t=0}^{t^*}\left| \ln \mathbb {E}[X(t;n)] - (\ln A - t/\tau ) \right| ^2, \end{aligned}$$

where *N* is the number of days in each period, that is, the number of booking curves series *X*(*t*) to be aggregated, and $t^*$ is a constant that gives the integral interval. The set of Fig. 6 and Table 7 shows the relationship between *CV* and *MSE* denote the degree of causes and the degree of results in the proposed model, respectively.

**Table 7:** Verify the proposed model’s causality by comparing the values *CV* and *MSE*, which imply one of the degrees of causes and results, respectively.

	2019		2020	
	*MSE*	*CV* %	*MSE*	*CV* %
Property I	$3.61\times 10^{-4}$	33.31	$5.96 \times 10^{-4}$	54.52
Property II	$1.48 \times 10^{-4}$	15.31	$6.02 \times 10^{-3}$	100.89
Property III	$2.17 \times 10^{-4}$	33.54	$2.28 \times 10^{-3}$	78.37
Property IV	$1.00 \times 10^{-4}$	36.80	$1.49 \times 10^{-4}$	51.88
Property V	$4.02 \times 10^{-4}$	35.57	$4.22 \times 10^{-4}$	62.95
Property VI	$6.99 \times 10^{-4}$	33.52	$2.26 \times 10^{-4}$	61.64

The value *CV* for each year is calculated from the series in Fig. 6. The correlation coefficient between *CV* and *MSE* is 0.79.

As for *CV* in the table in Table 7, comparing 2019 and 2020, they were 1.5 to 6.5 times larger in 2020 through all properties. Looking more closely, the rate of increase from the previous year at properties II and III was more significant than at the other properties. This result reflects the fact seen in Table 4. As for the exponential degree, *MSE* in properties II and III in 2020—the environments with the highest *CV*— were one digit order larger than the other environments. Although we see a case with inverse correlation, such as the property VI, we confirm that changes in *CV* were positively linked to changes in *MSE*. Finally, the correlation coefficient between *CV* and *MSE* calculated from the results in the table was 0.79. This result of the correlation between the “degree” of a homogeneous environment and the exponential property of average booking curves yields sufficient evidence for the causality of the model that the HDSBA leads to the exponential law. Note that we visualize each property’s actual average booking curves for 2019 and 2020 in the same way as Supplementary Fig. S1 in the supplemental document.

Furthermore, as mentioned in Section “The data analyzed”, we did not aggregate booking curves for chartered days to exclude group travel customers having different booking patterns from usual customers, whose objectives consist of escorted tours or school excursions. Thus, we discuss the case with chartered days in the supplemental document and explain one of the limitations of the ABCDEF law; we show that the high ratio of group customer results does not satisfy the ABCDEF law according to mixed (non-homogeneous) booking patterns. That implies that “customer attributes” can become one of the elements defining a homogeneous demand-supply environment, in addition to the economic background.

As described above, Section “A universal statistical law: average booking curves draw exponential functions” has presented the evidence for the ABCDEF law from three perspectives; data validation, modeling in the statistical physics framework under the HDSBA, and empirical justification for the causality of the model. It is highly expected that the average booking curves, a time series inherent in perishable asset industries, can universally follow the exponential law regardless of the economic environment or industry with comprehensive justification.

## Discussion

This section discusses how the ABCDEF law can contribute to the industry’s future development. One of the advantages of the application is that it is industrially unlimited. Some forecasting or dynamic pricing applications that can be applied in various perishable asset industries have already been proposed^21,45^.

However, the ABCDEF law provides a booking curve, mainly used as a daily means of forecasting, with another usefulness. The following section explains quantitative and informative statistics indicating people’s booking patterns based on the ABCDEF law.

### A new statistic for measuring booking window shifts

As seen in Section “Literature review”, a conjoint analysis for hotel managers reported that timing is the most important factor in pricing. In addition, complex shifts in the booking window have provided challenges for RM professionals to adapt continuously. In other words, recent changes have let us pay more attention to the booking window shifts to maintain or improve the performance of RM.

Here, we have the following proposal. While the average lead day of bookings has been used as a statistic to measure booking window shifts, we are unsure whether this is a helpful statistic. That is because the average lead day of bookings is less informative; in other words, it only has limited information about the entire booking horizon to determine the appropriate timing to take RM measures. Besides, an average lead day cannot reflect the bookings at the deadline day ($t=0$) in which the most number of bookings emerge according to the ABCDEF law.

Based on this statement, we believe that a “booking pace baseline statistic”—a new statistic that quantifies how fast bookings build up under the current demand-supply environment—can give RM practitioners more information about people’s booking patterns, that is, booking window shifts, than a conventional average lead day of bookings.

In addition, we propose that $\beta$, one of the parameters inherent in the ABCDEF law, can serve as a sample of “booking pace baseline statistics.” While the parameter $\beta$ reflects the environment surrounding the property in the ABCDEF law, it also illustrates patterns in people’s booking horizons, which can be recognized from the shape of the function. Therefore, it has more information about the build-up pace over the whole booking horizons. Table 8 compares the characteristics of average lead days and “booking pace baseline statistics” based on $\beta$. The booking pace baseline statistics characterized by $\beta$ is considered superior to the conventional statistics in terms of the amount of information about the entire time series. The unique booking pattern for whole booking horizons periodically derived is expected to be a practical measure for RM professionals in making strategies, including pricing, in which the timing is the crucial factor.

**Table 8:** Comparison of the characteristics of booking pace baseline statistics given by $\beta$ and average of lead days of bookings, which is the conventional statistics for evaluating booking window shifts.

	Statistics for measuring booking window shift	
	Average of lead days	$\beta$: booking pace baseline statistics
Versatility	$\bigcirc$	$\bigcirc$
Information about characteristics of time series	Less: just one point of time	More: whole booking horizons

## Conclusion

This study is based on actual sales data for the two years of 2019 and 2020 from six properties in multiple industries, including the hotels and car rental fields. We investigated macroscopic aspects of booking curves, considering the difference in the economic environment characterized before and during the COVID-19 epidemic. We justified the universality of the ABCDEF law from the following three perspectives: data validation, modeling in the statistical physics framework, and empirical justification for the causality of the model. In particular, in the modeling section, we introduced an assumption for the environment surrounding the property (a homogeneous demand-supply bath assumption: HDSBA) and explained the mechanism that average booking curves follow exponential functions. In the empirical justification for the causality section, we reinforced the basis by investigating the correlation of two “degrees” defining a homogeneous environment and exponential property, respectively.

The ABCDEF law provides a booking curve with its usefulness besides daily forecasting in RM. The new measure for booking window shift, “booking pace baseline statistics,” characterized by the parameter in the ABCDEF law, provides RM practitioners with practical information about people’s booking patterns in the current demand-supply environment. It is expected to be helpful in RM in various perishable asset industries, based on selling products at the right price and timing since people’s booking windows have changed due to environmental shifts.

### Supplementary Information

Supplementary Information.

## Data Availability

The datasets analyzed during the current study are available in the Kyoto University Research Information repository, https://doi.org/10.57723/276374.
