# Supplementary material for: Average booking curves draw exponential functions
Source: Sci Rep. 2023 Sep 22;13:15773. doi: 10.1038/s41598-023-42745-3 (PMC10517154; doi:10.1038/s41598-023-42745-3)
Supplement: Supplementary file 1 — Supplementary Information. [file 41598_2023_42745_MOESM1_ESM.pdf]

Supplementary document for "Average Booking Curves Draw Exponential Functions"

Masaru Shintani[1,2,*] **and Ken Umeno**[1]

[1]Kyoto University, Graduate School of Informatics, Kyoto, 606-8501, Japan
[2]FORCIA, Inc., Research and Development Department, Tokyo, 160-0022, Japan
[*]shintani.masaru.28a@kyoto-u.jp

1 Visualized data validation for 2019 and 2020 with average booking curves

In Sec. 5 in the main paper, to verify the causality of the proposed model, we investigate the correlation of the following two variables: the coefficient of variation CV, which represents the quantitative degree of a homogeneous demand-supply environment, and the mean squared error MSE, which is defined as fitting deviation between exponential functions and average booking curves. Then we show the positive correlation (the coefficient of correlation is 0.79) in CV and MSE. In this supplementary document, we visualize each property's average booking curves for 2019 and 2020. The average booking curves deviate from exponential functions according to large MSE, especially in properties II and III in 2020. As for their environments, the most significant low demand (see in Table 4 in the main paper) occurred mainly during the state of emergency period, which caused a considerable bias in last-minute bookings. Due to the unstable demand condition, we confirm how the average booking curves deviate from exponential functions.

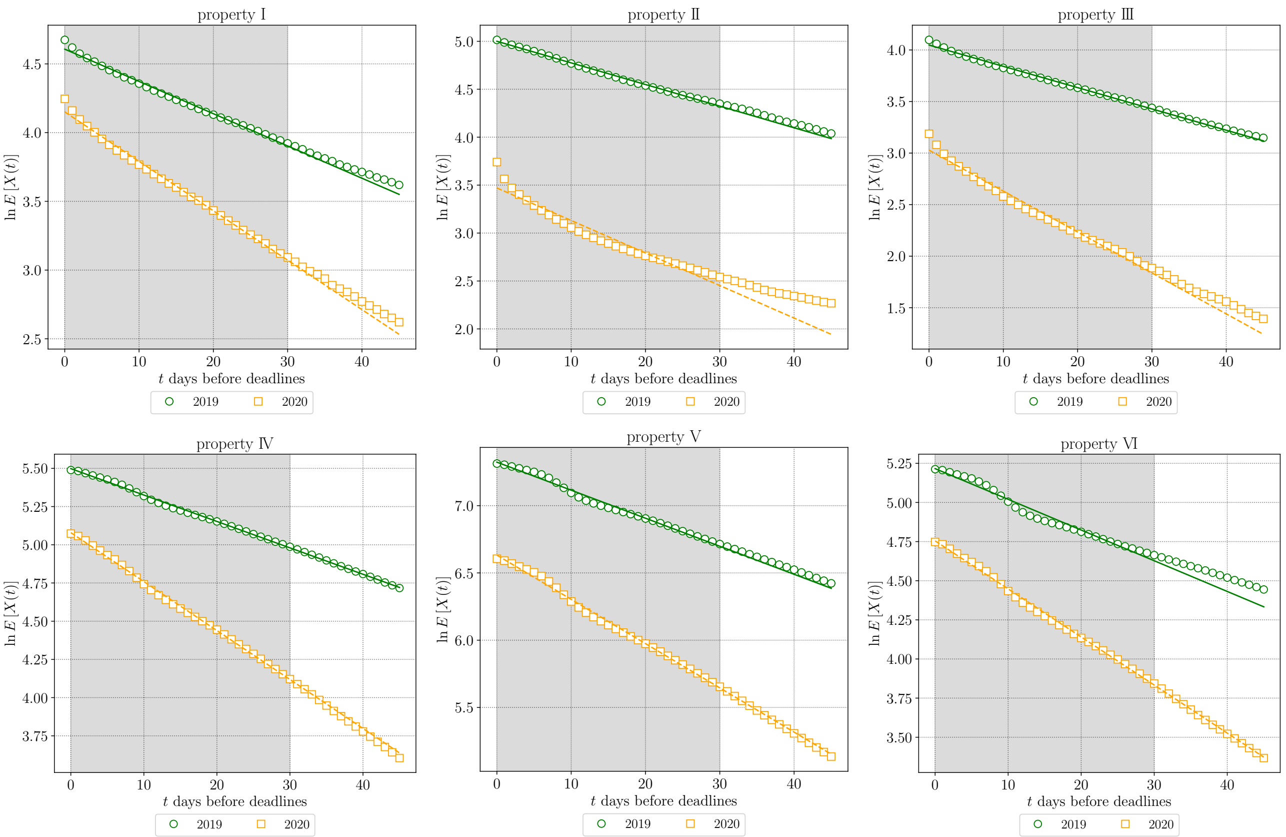

Supplementary Figure S1. Figures show average booking curves and exponential functions based on annual data for 2019 and 2020 and provide visual information for variables MSE in the main paper.

2 Investigation of booking curves, including group travelers in property I

This supplementary document visualizes the limitation of the ABCDEF law when including group travelers with unusual booking patterns in property I.

In the hotel industry, group travelers whose objective includes escorted tours or school excursions have a different booking pattern from individual travelers; property I has mainly individual travelers and sometimes receives group travelers. Figure S2 illustrates the information about the group ratio in property I in 2019 and defines five types of aggregation patterns. Figure S3 shows that average booking curves with a high rate of group guests result in not drawing exponential functions. Note that case ii is the same subject as shown in the main paper, and case i shows high exponential property as with case ii.

These results imply that one of the limitations of the ABCDEF law results from the mixture of booking behaviors. In other words, customer attributes, which generate differences in booking behavior patterns, can become one of the elements defining a homogeneous demand-supply environment.

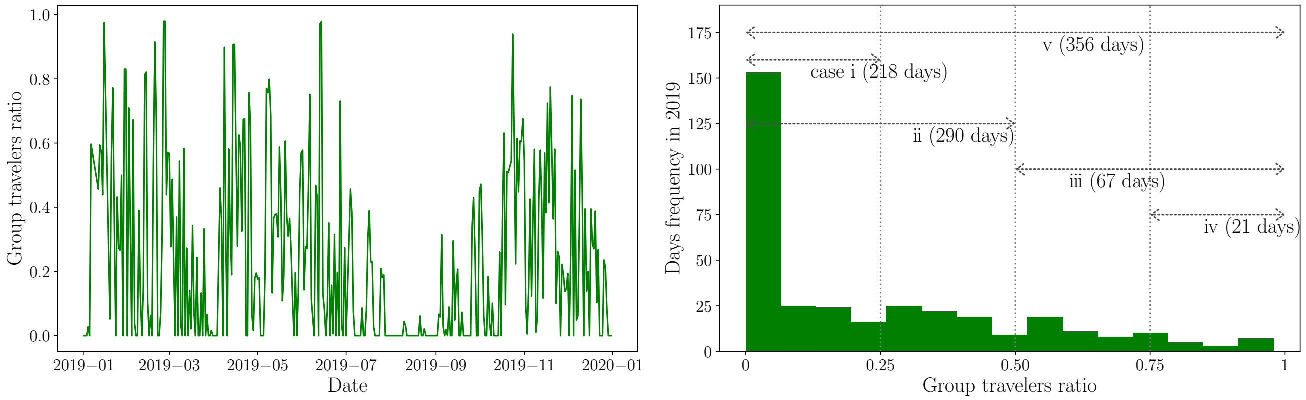

Supplementary Figure S2. The information about group travelers ratio in property I in 2019. The left figure shows the ratio of group travelers in terms of used rooms unit in 2019. The right figure illustrates the histogram of the ratio shown in the left figure. The labels i, ii, iii, iv, and v, represent the aggregating targets for the identical and group travelers, and case ii corresponds to the subject in the main paper.

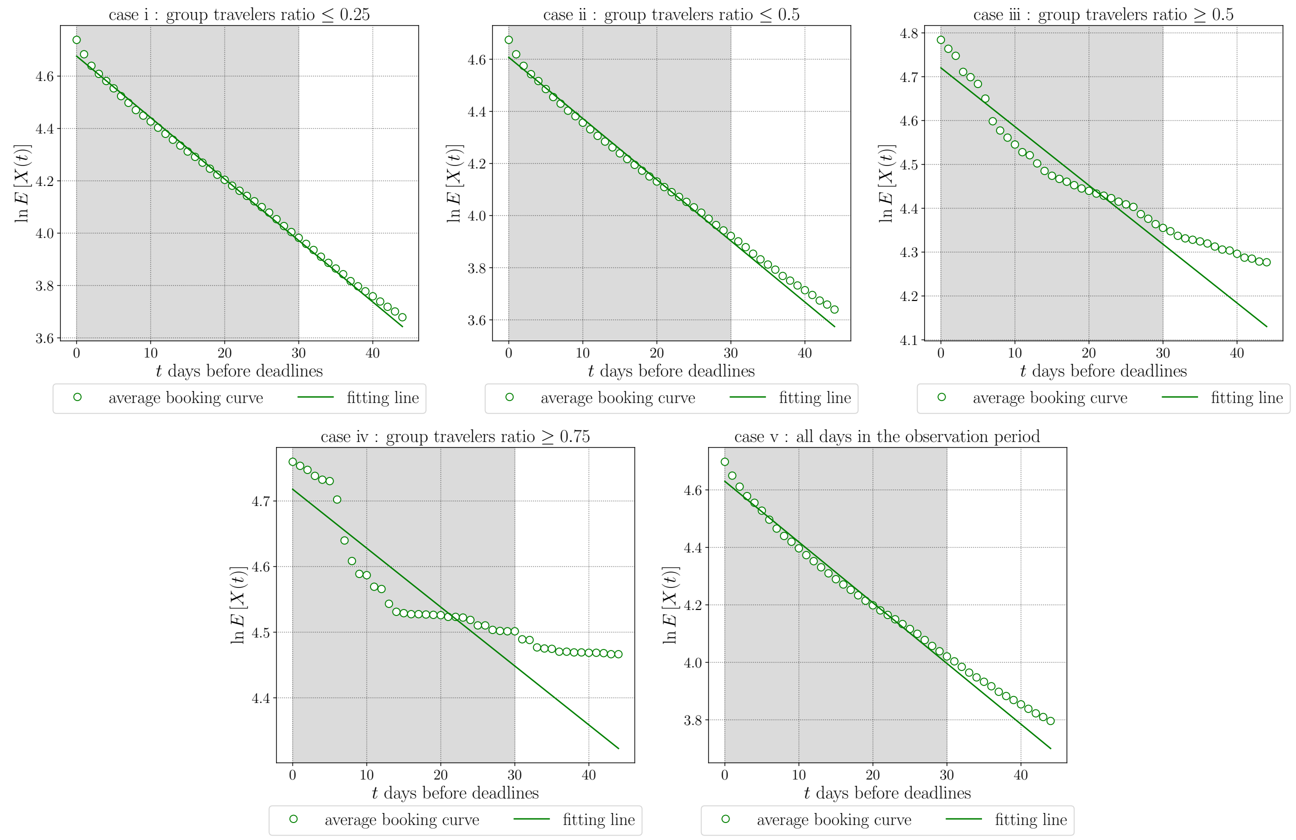

Supplementary Figure S3. This figure illustrates the average booking curves in each aggregate pattern of i, ii, iii, iv, and v, defined in Fig. S2.
